# Supplementary material for: Molecular Manipulation of the miR396 and miR399 Expression Modules Alters the Response of Arabidopsis thaliana to Phosphate Stress
Source: Plants (Basel). 2021 Nov 24;10(12):2570. doi: 10.3390/plants10122570 (PMC8706208; doi:10.3390/plants10122570)
Supplement: Supplementary file 1 [file plants-10-02570-s001.zip › plants-1447838-supplementary.pdf]

Molecular manipulation of the miR396 and miR399 Expression Modules Alters the Response of *Arabidopsis thaliana* to Phosphate Stress

Joseph L. Pegler [1], Duc Quan Nguyen [1,2], Jackson M.J. Oultram [1], Christopher P.L. Grof [1,†] and Andrew L. Eamens[1,3,4,†,*]

[1] Centre for Plant Science, School of Environmental and Life Sciences, Faculty of Science, University of Newcastle, Callaghan, NSW 2308, Australia; joseph.pegler@newcastle.edu.au (J.L.P.); ducquan.nguyen@uon.edu.au (D.Q.N); jackson.oultram@uon.edu.au (J.M.J.O); chris.grof@newcastle.edu.au (C.P.L.G).
[2] Institute of Genome Research, Vietnam Academy of Research and Technology, 18 Hoang Quoc Viet Str., Cau Giay, Hanoi 100000, Vietnam.
[3] School of Science, Technology and Engineering, University of the Sunshine Coast, Maroochydore, Queensland, Australia, 4558.
[4] School of Chemistry and Molecular Biosciences, The University of Queensland, Brisbane, Queensland, Australia, 4072.
* Correspondence: a.eamens@uq.edu.au

Table S1. The DNA oligonucleotide sequences of the stem-loop primers used for the reverse transcription of miR396- and miR399-specific cDNAs are provided below in the Table S1 (denoted by RTSL). Post synthesis of a miR396- and miR399-specific cDNA, miRNA-specific forward primers (denoted by RTF in the below Table) together with a generic reverse primer (denoted by SLR in the below table (underlined sequence of the RTSL primer identifies the binding site for the generic reverse primer)) were used to quantify miR396 and miR399 abundance. Also provided below in Table S1 is the sequence of the DNA oligonucleotide used as either the forward (denoted by RTF in the below Table) or reverse (denoted by RTR in the below Table) primer to quantify the expression of either miR396 or miR399 target genes, or a cohort of PO_4-related high molecular weight RNA transcripts. The snoRNA, *snoR101*, was used to normalize miRNA abundance across the different plant lines assessed in this study. The reference gene, *UBI10* (*AT4G05320*), was used to normalize the expression of each analyzed high molecular transcript.

Targeted Sequence	Primer Name	Oligonucleotide sequence (5' to 3')
**DNA oligonucleotides used to quantify sRNA abundance**		
**miR396**	**p396-RTSL**	GTCGTATCCAGTGCAGGGTCCGAGGTATTCGCACTGGATACGACAAGTTC
	**p396-RTF**	GCGCGTTCCACAGCTTTCTTGAAC
**miR399**	**p399-RTSL**	GTCGTATCCAGTGCAGGGTCCGAGGTATTCGCACTGGATACGACCAGGGC
	**p399-RTF**	GCATGCCAAAGGAGATTTGCCCTG
**miRNA stem-loop oligo**	**pSLR-Generic**	CCAGTGCAGGGTCCGAGGTA
**snoR101**	**psnoR-RTF**	CTTCACAGGTAAGTTCGCTTG
	**psnoR-RTR**	AGCATCAGCAGACCAGTAGTT
**DNA oligonucleotides used to analyze gene transcript expression**		
***GRF1* (*AT2G22840*)**	**pGRF1-RTF**	CGTCGCATAAACAAGCCTCG
	**pGRF1-RTR**	ATTTCAGCTCTTCGGGCCAA
***GRF2* (*AT4G37740*)**	**pGRF2-RTF**	CTTGGCCTGAAGAGCTGACA
	**pGRF2-RTR**	GTGTGTGGAGGAAGGGGATG
***GRF3* (*AT2G36400*)**	**pGRF3-RTF**	CCATACGAGTCCCACATCGG
	**pGRF3-RTR**	CTGAGCTCATGGGGCTTGAA
***GRF7* (*AT5G53660*)**	**pGRF7-RTF**	CATCCCCCACCGTTAGATCG
	**pGRF7-RTR**	TGCTTCCATGCTTCCGACAT

DNA oligonucleotides used to analyze gene transcript expression (continued)		
GRF8 (*AT4G24150*)	pGRF8-RTF	GCTGCTGTGACTGTAGCAGA
	pGRF8-RTR	CTCATGCCATTGAGCTTCGC
GRF9 (*AT2G45480*)	pGRF9-RTF	CTCACATGAGAATGCCGGGT
	pGRF9-RTR	ATCAGAAACTCGGGGCAGTG
MIR399A (*AT1G29265*)	p399A-RTF	AGGGTAAGATCTCTATTGGCAGGAAAC
	p399A-RTR	GCAGAAGAATTACAGGGCAAATCTCC
PHO2 (*AT2G33770*)	pPHO2-RTF	ACCGTTTCTCATCAAGGCGT
	pPHO2-RTR	GTGCCCGTCCACCATAAGAA
PHR1 (*AT4G28610*)	pPHR1-RTF	AAACCAACCCGGCGATTCA
	pPHR1-RTR	CAGCCCATTCATGCCAATCACTT
PHT1;4 (*AT2G38940*)	pPHT1-4-RTF	TGTGCCGGCCGAAATCT
	pPHT1-4-RTR	TTGCTCCTAATTTTCCTGATGCT
PHT1;8 (*AT1G20860*)	pPHT1-8-RTF	CCCGAAGTAAACCGTATGAGAA
	pPHT1-8-RTR	AATACGTCACCAAGATTCCAGCAA
PHT1;9 (*AT1G76430*)	pPHT1-9-RTF	TGGAGCTGCAGGGAAGTTTG
	pPHT1-9-RTR	ATCTGGAAAACCGTCCTCTTCAT
UBI10 (*AT4G05320*)	pUBI10-RTF	GGCCTTGTATAATCCCTGATGAATAAG
	pUBI10-RTR	AAAGAGATAACAGGAACGGAAACATA
